# Senescent *Eimeria acervulina* Oocysts Maintain Transcriptional Activity During Extended Refrigerated Storage and Differentially Express Characteristic Genes

**DOI:** 10.3390/microorganisms14051116

**Published:** 2026-05-14

**Authors:** Matthew S. Tucker, Doaa Naguib, Celia N. O’Brien, Christina Yeager, Benjamin M. Rosenthal, Mark C. Jenkins, Asis Khan

**Affiliations:** 1 Animal Parasitic Disease Laboratory, Beltsville Agricultural Research Center, Agricultural Research Service, United States Department of Agriculture, Beltsville, MD 20705, USA; doaa.hassan@usda.gov (D.N.); cnobrien26@yahoo.com (C.N.O.); christina.yeager@usda.gov (C.Y.); benjamin.rosenthal@usda.gov (B.M.R.); mark.jenkins@usda.gov (M.C.J.); asis.khan@usda.gov (A.K.); 2 College of Medicine, Lake Erie College of Osteopathic Medicine, Bradenton, FL 34211, USA

**Keywords:** *Eimeria*, RNA sequencing, *Cyclospora*, viability, oocyst, senescence, detection, food safety, vaccine

## Abstract

Enteric coccidian parasites harm agriculture and human health. Infectious, sporulated parasites eventually senesce. Here, we examined transcriptional changes in sporulated oocysts of *Eimeria acervulina* stored for 4–30 months at 4 °C. Precipitous decline in RNA abundance and transcription followed an interval of stability. Sixty constitutively expressed genes each contributed > 1000 transcripts per million (TPM) throughout, including a serine protease inhibitor, surface antigen genes, a cation-transporting ATPase, an oocyst wall protein, a zinc finger DHHC domain-containing protein, and highly expressed hypothetical proteins with no known function. Strikingly, ~82% of 6867 annotated genes underwent differential expression when comparing freshly sporulated parasites to those held for 30 months; nearly one-third of these underwent significant expression change. In freshly sporulated oocysts, 86 significantly DEGs exceeded 1000 TPM; these encoded heat shock proteins, lactate dehydrogenase, glucose-6 isomerase, and various hypothetical proteins. The oldest parasites expressed 66 DEGs, including many ribosomal subunits, a haloacid dehalogenase-like hydrolase domain-containing protein, and various hypothetical proteins. Taken together, these findings helped us to identify markers of mature parasites that remain relatively abundant in the transcript pool as oocysts age and identify other transcripts (e.g., ribosomal RNA) that increase in their relative abundance even as RNA abundance declines in senescent parasites.

## 1. Introduction

Coccidia cause enteric disease in people (i.e., *Cyclospora cayetanensis*), and closely related apicomplexan parasites cause poultry coccidiosis causing an estimated USD 14 billion in economic harm annually [1]. One of seven species of *Eimeria* typically causes this disease in chickens; each infects distinct regions of the intestinal tract. Among these species, *E. maxima*, *E. tenella*, and *E. acervulina* impose a huge economic burden on the poultry industry globally [2]. Control of poultry coccidiosis involves vaccines incorporating low doses of live, precocious oocysts, consisting of multiple species [2,3,4]. There has been renewed interest in the application of live oocyst vaccines in commercial broilers, prompted by recent regulatory actions that prohibit certain antibiotic growth promoters and the growing global consumer demand for reduced pharmaceutical use in animal husbandry. However, the successful implementation of an immunization program using live oocyst vaccines necessitates the development of a consistent vaccine formulation based on viable sporulated oocyst counts. The absence of precise tools for conducting viability assays significantly impedes the effectiveness of these immunization programs.

Coccidian parasites are passed from their hosts as unsporulated oocysts; these must undergo development (sporulation) to become infectious [5]. Sporulation varies with environmental conditions including temperature, oxygenation, and humidity. Sporulation rates vary among species; under optimal laboratory conditions, most mature by 48 h [6,7,8]. *E. acervulina* can become infectious in as few as 7 h [8]. *C. cayetanensis*, by contrast, requires up to 2 weeks to completely sporulate [9].

Few studies have examined the transcriptomes of sporulating oocysts of *Eimeria;* none yet exist for *C. cayetanensis*. We previously employed RNA sequencing (RNA-Seq) to characterize sporulation in *E. acervulina* [10] and *E. tenella* (unpublished data), two strains of *E. maxima* differing in their pathogenicity [11]; doing so identified highly expressed genes throughout sporulation in one or more parasite species. We also identified genes that undergo differential expression as oocysts mature. Such biomarkers may prove useful in distinguishing non-viable from viable oocysts and complement emerging techniques (e.g., AI viability assessment of coccidian oocysts [12]), assisting diagnostic, public health, and vaccine assessments. As a model, these data also provide guideposts for predicting genes that may similarly govern maturation in *C. cayetanensis*, because many of the genes orchestrating development in species of *Eimeria* have direct homologs in this human parasite. Thus, illuminating such developmental processes promotes the goals of animal health and food safety.

Here, for the first time, we examined gene expression in aging coccidian parasites. We were motivated to understand regulators of extrinsic development whose disruption might slow or stop development to the infectious state. Doing so would benefit public health surveillance and livestock husbandry. We also hoped to identify biomarkers characteristic of dying and dead parasites and elucidate transcriptional pathways differentiating non-viable from viable oocysts. To do so, we took advantage of sporulated parasites held at room temperature, and at 4 °C, for over 30 months. We previously documented when each species loses infectiousness (by assaying them at 3-month intervals via in vivo infection and in vitro excystation) [13], finding that in vitro excystation did not correlate consistently with oocyst infectivity. This work established that *E. tenella* and *E. maxima,* held at 4 °C, lose the ability to infect chickens after 9 and 12 months, respectively, but that *E. acervulina* remains infectious through 27 months.

We analyzed the pool of transcripts in these successively older cohorts of sporulated *E. acervulina* oocysts, stored at 4 °C. We compared the transcriptomes of aged cohorts to one another and to freshly propagated sporulated and unsporulated oocysts. We included oocysts stored from 4 to 30 months, knowing that the oldest cohort (30 months) failed to infect chickens when administered at doses previously reported [13]. Guided by previous RNA sequencing of *E. acervulina,* we aimed to examine how previously posited biomarkers are expressed while oocysts decay and illuminate, more generally, the transcriptional dimensions of senescence.

## 2. Materials and Methods

### 2.1. Ethics Statement

Chickens were used to produce fresh oocysts of *E. acervulina* following protocol 25-05 approved by the BARC Institutional Animal Use and Care Committee, United States Department of Agriculture. We examined cohorts of aged oocysts stored at 4 °C as previously described [13]. The chickens utilized in experiments exhibited no outward signs of severe disease over the course of the study. After each experiment’s conclusion, chickens were humanely euthanized, and all efforts were made to minimize animal suffering.

### 2.2. Parasites

To obtain fresh oocysts, we employed methods described in [13]. Chickens (HR708 broilers, Longneckers Hatchery, Elizabethtown, PA, USA) were infected with 1.5 × 10^5^ sporulated oocysts of *E. acervulina*. On day 6 post-inoculation, feces from the birds were collected. Unsporulated oocysts were isolated from chicken feces via flotation on a saturated salt solution and then washed 3–4 times with water. The final oocyst preparation was resuspended in 2% *v*/*v* potassium dichromate (Sigma Aldrich, St. Louis, MO, USA) in a sterile 1 L flask. An aliquot of unsporulated oocysts was reserved and then processed as described below. To obtain freshly sporulated oocysts, unsporulated oocysts were incubated in a 29 °C shaking water bath aerated using an aquarium pump for 24 h.

The aged *E. acervulina* oocysts (from 4 to 30 months) utilized in this study were originally reported in [13]; these were propagated, processed, and sporulated as described above, followed by storing in 500 mL bottles containing 2% (*v*/*v*) potassium dichromate at 4 °C.

We selected several parasite cohorts for this study: freshly isolated, unsporulated oocysts (denoted here as unsporulated) and sporulated oocysts (denoted here as 0 months), and seven batches of aged parasites held at 4 °C. The aged parasite cohorts were held for 4, 8, 13, 18, 25, 29, and 30 months prior to RNA extraction. For each oocyst cohort, aliquots were removed from storage containers and transferred to 50 mL centrifuge tubes. Oocysts were centrifuged at 2400× *g* for 5 min at 4 °C and washed with deionized water to remove excess potassium dichromate. The oocysts were then incubated with 6% sodium hypochlorite for 15 min with rocking agitation at room temperature to remove exogenous microbial contaminants. After bleach treatment, samples were washed several times with deionized water to remove residual bleach. Oocysts were enumerated by microscopy using a hemacytometer. Each cohort contained at least 2 × 10^6^ oocysts at the end of the procedure. After washing, oocysts were resuspended in TRIzol (Thermo Fisher Scientific, Waltham, MA, USA) and frozen at −80° C.

### 2.3. Microscopy

Oocysts were examined via microscopy for morphological changes throughout storage at 4 °C. Prior to taking photomicrographs, aliquots of oocysts were transferred into a 1.5 mL microcentrifuge tube and centrifuged at 8000 rpm for 2 min. The oocyst pellets were washed twice with water to remove excess potassium dichromate, and the final pellets were resuspended in 100–200 µL water before visualization. Images of representative oocysts at each time point were captured at 1000× total magnification using a Leica DM750 compound microscope (Leica Microsystems Inc., Deerfield, IL, USA) fitted with a Path4K camera and InFocus software version x64, 4.12.24100.20231210 (I. Miller Microscopes, Feasterville, PA, USA).

### 2.4. Sample Preparation for RNA-Seq

Oocysts frozen in TRIzol were thawed on ice and transferred to a 15 mL Wheaton Potter-Elvehjem Tissue Grinder tube (DWK Life Sciences Millville, NJ, USA). Each cohort was processed separately. The suspension was ground using a Teflon pestle attached to a Wheaton overhead stirrer in a total volume of 1.5 mL for 5 cycles of 25 grinds each. The samples were periodically examined by microscopy to monitor the progress of oocyst and sporocyst breakage. Homogenates were transferred into 2 mL RNase-free microcentrifuge tubes and centrifuged at 10,000 rpm for 10 min at 4 °C. The supernatants were transferred to new tubes and mixed with 0.3 mL chloroform, then incubated at room temperature for 3 min. The tubes were centrifuged at 13,500× *g* for 15 min at 4 °C. The upper phase was transferred into a new 1.5 mL RNase-free microcentrifuge tube and mixed with 0.5 mL isopropanol. Tubes were centrifuged at 12,000× *g* for 10 min at 4 °C, after which supernatants were decanted. Pellets were resuspended in 1 mL of 75% ethanol, and tubes were centrifuged at 7500× *g* for 5 min at 4 °C. The supernatant was removed, and the wash was repeated two more times. RNA pellets were resuspended in molecular grade DEPC-treated water.

Total RNA was quantified using a Qubit 3.0 fluorometer and a Qubit RNA High Sensitivity kit (Thermo Fisher Scientific), and the quality (RNA Integrity Number [RIN]) was assessed using a Bioanalyzer 2100 with software version B.02.12 (SR2) and RNA 6000 Nano kit (Agilent Technologies, Santa Clara, CA, USA) as well as a Tapestation 4150 with software version 4.1.1 and High Sensitivity RNA Screen Tape and reagents (Agilent Technologies). The RNA samples were frozen at −80°C until further use. Total RNA was DNase-treated with an Invitrogen Turbo DNA-free kit (Thermo Fisher Scientific). Owing to low RNA yields, we pooled multiple batches at times to ensure adequate concentrations of DNase-treated RNA for performing replicate RNA-Seq assays. Pooled DNase-treated RNA for each cohort was checked again by Qubit and Tapestation/Bioanalyzer prior to sequencing. RIN values of such RNA pools mirrored their values prior to pooling.

### 2.5. cDNA Library Construction and RNA-Seq

Library construction and RNA-Seq followed previous methods [11], with some exceptions. For each developmental stage and age cohort, coding mRNA libraries were produced from each of three technical replicates of oocysts. Approximately 200 ng of total RNA was used as input with the Illumina Stranded mRNA Prep ligation kit (Illumina, San Diego, CA, USA) to prepare double-stranded cDNA libraries. Three cDNA libraries were produced from each starting RNA sample. In most libraries (except those prepared from RNA extracted at 0 (unsporulated and sporulated oocysts), 4, and 8 months), unbound Illumina adapter fragments (~150 bp) were abundant. To remove these, we employed size selection through gel electrophoresis and compartment elution, using a Blue Pippin instrument (Sage Science, Beverly, MA, USA). Specifically, we used 2% agarose gel cassettes, dye free with internal standards, to select 200–600 bp fragments.

The final cDNA libraries for each replicate were quantified using a Qubit dsDNA High Sensitivity kit (Thermo Fisher) or Tapestation High Sensitivity DNA ScreenTape and reagents (Agilent Technologies), and the size distribution of libraries was characterized by using a Bioanalyzer 2100 High Sensitivity DNA kit (Agilent Technologies) and Tapestation High Sensitivity DNA ScreenTape and reagents. The average library size ranged from 324 to 379 bp among the samples; samples were normalized for stoichiometric balance. Blue Pippin size selection reduced library molarity (minimum library concentration = 56 pM) compared to RNA preparations (from cohorts 8 months or younger) requiring no such step. All libraries were then sequenced on an Illumina MiSeq (Illumina, San Diego, CA, USA). The indexed, pooled libraries were first run on a 300-cycle Illumina MiSeq Reagent Nano Kit v2 nano (151 × 2 paired-end reads) at 6 nM loading concentration, to check read distribution; then, a final run utilized a 600-cycle MiSeq Reagent Kit v3 at 15 pM loading concentration.

After the run, paired-end sequencing reads were imported into Geneious Prime 2024.07 (Biomatters Inc. Ltd., Auckland, New Zealand), followed by both quality-trimming and adapter-trimming using the package BBDuk (version 38.84) [11]. The trimmed reads were aligned with the *E. acervulina* Houghton reference strain (annotated NCBI assembly EAH001 [14]) using the Geneious RNA mapper (https://www.geneious.com). The expression for each mapped sample was estimated as transcripts per million (TPM), calculated by Geneious as (CDS read count × mean read length × 10^6^)/(CDS length × total transcript count). Data tables with raw mapped read counts, nucleotide and protein sequences, and TPM per gene were exported from Geneious as .csv files and further analyzed in Microsoft Excel (Redmond, WA, USA) to calculate descriptive statistics of mapped reads and TPM for the sample replicates. Data analysis and visualization were aided by Daniel’s XL Toolbox add-in for Excel, version 7.3.4, by Daniel Kraus, Würzburg, Germany (www.xltoolbox.net).

### 2.6. Comparing Gene Expression Among Oocyst Cohorts

Correlation (Pearson’s) of the normalized RNA-Seq mapped reads (log_2_ transformed) among age cohorts and replicates was depicted as a heatmap constructed in RStudio 2024.04.2 Build 764 (R version 4.4.2) using the packages ggplot2 version 3.5.1 [15] and reshape2 version 1.4.4 [16]. To examine variance in transcribed genes, mean TPM for each gene in each of three technical replicates was calculated and imported into RStudio. A filter retained genes with >0.01 TPM in all cohorts; TPM was then log_2_ transformed before calculating variance. The top 50 genes were selected to display in a heatmap. To visualize hierarchical clustering, heatmap.2 (gplots version 3.1.3.1 [17]), pheatmap version 1.0.12 [18], and ComplexHeatmap version 2.20.0 [19] packages in R were used to represent the matrix of Euclidean distances.

### 2.7. Differential Gene Expression Through Time

We used the DESeq2 package [20], as implemented in Geneious Prime 2024.07 (https://www.geneious.com), to estimate pairwise differential gene expression in 0- and 30-month oocyst cohorts, incorporating three technical replicates per group. Data were exported from Geneious as .csv files and gene log_2_ ratios; adjusted *p*-values were used for downstream analyses. We used thresholds of >1.5 or <−1.5 log_2_ fold change (FC) with an adjusted *p*-value < 0.05 to identify significantly differentially expressed genes (DEGs). Differential expression could not be determined for some of the 6867 annotated genes, because DESeq2 [20] does not return log_2_ FC and adjusted *p*-values when all replicates have zero read counts for a gene (this occurred for 508 genes). Adjusted *p*-values were also not determined for some genes that did have log_2_ FC because a gene was filtered by automatic independent filtering for having a low mean normalized read count (an additional 737 genes). Volcano plots depicting the log_2_ FC vs. −log_10_ adjusted *p*-values (Adj. *p*-value) for DEGs were constructed in the R package Enhanced Volcano version 1.22.0 [21]. Correlation plots comparing log_2_ FC of DEGs between strains were constructed in the R package ggplot2.

### 2.8. Homology Searching and Functional Genomics Analysis

Functional information on genes of interest was derived from the annotations available for the *E. acervulina* Houghton reference strain in NCBI (assembly EAH001) and ToxoDB v68 [22] (www.toxodb.org, accessed 22 February 2026). To elucidate the additional functions of genes, we utilized the functional analysis module in OmicsBox 3.2.4 (BioBam Bioinformatics, Valencia, Spain). Our Blast2GO® [23] (https://www.biobam.com/omicsbox/, accessed 11 April 2026) workflow (for Phylum Apicomplexa or *Eimeria* spp.) and settings were previously described in [11]. Additionally, the eggNOG mapper (evolutionary genealogy of genes: Non-supervised Orthologous Groups; version 2.1.0 with eggNOG 5.0.2 [24]) inferred the orthology relationships, gene evolutionary histories, and functional annotations to improve the Blast2GO® sequence characterization. We used KEGG (Kyoto Encyclopedia of Genes and Genomes [25]) in the pathway analysis settings, which enabled biochemical pathway enrichment analysis, linking pathways via Enzyme Commission (EC) numbers. The Blast2GO® results and lists of GO annotation terms and KEGG pathways were exported from OmicsBox and analyzed further in Microsoft Excel. Additional information for gene homologs and orthologs/paralogs and KEGG pathways (exact EC matches only) was found by searching the data sets in ToxoDB.

### 2.9. Quantitative PCR and Digital Quantitative PCR to Validate RNA-Seq

We employed real-time quantitative PCR (qPCR) and digital PCR (dPCR) to validate estimates of gene expression from genes selected either for their strong, constitutive expression (*EAH_00004110*) or because of their markedly distinct relative abundance in the transcript pools derived from new (0 month) or old (30 month) cohorts of sporulated oocysts (*EAH_00020350*, *EAH_00049190*). To do so, we prepared individual qPCR reactions using SsoAdvanced Universal SYBR Green Supermix (Bio-Rad, Hercules, CA, USA), with 200–300 nM of each primer (Table S1), and 1 µL of diluted double-stranded cDNA sequencing library to a total volume of 10 µL. All primers (Table S1) were designed using NCBI Primer-BLAST [26] (https://www.ncbi.nlm.nih.gov/tools/primer-blast/, accessed 3 May 2024) and synthesized by Integrated DNA Technologies (Coralville, IA, USA). cDNA template was derived from the sequencing library prep described above. The 30-month sample had undergone Blue Pippin purification. Beta tubulin expression (*EAH_00006010*) served as a reference to normalize expression of other assayed transcripts. On each assay plate, we included triplicate reactions of the target gene and for Beta tubulin for each technical replicate of RNA prepared from 0-month and 30-month cohorts. Gene expression was estimated for Beta tubulin and target genes after averaging the mean Cq values for each technical replicate. The fold change (FC) in expression between 0- and 30-month cohorts was estimated using an efficiency-corrected relative expression method [27]. The log_2_ FC of genes between groups by RNA-Seq (determined by DESeq2) was then compared to the log_2_ expression determined from qPCR (mean of three trials).

Digital qPCR utilized the cDNA templates described above and included the additional cohorts 25 and 29 months. Individual digital qPCR reactions were prepared with 3X QIAcuity EvaGreen Mastermix (Qiagen, Germantown, MD, USA) using 0.4 μM of each primer and 2 µL diluted cDNA in a total volume of 40 µL. Reactions were carried out on a QIAcuity One digital PCR instrument (Qiagen) using reaction conditions of an initial denaturation at 95 °C for 2 min, followed by 40 cycles of denaturation at 95 °C for 15 s, annealing at 60 °C for 15 s and extension at 72 °C for 15 s, with a final incubation at 40 °C for 5 min. Absolute expression graphs depicting partition amplification and thresholds were set automatically by the QIAcuity software suite version 3.2. The nano-partitions were identified as positive or negative according to the fluorescence intensity values. The proportion of positive partitions determined the copy number by applying Poisson statistics in the QIAcuity software as described by [28]. The total number of copies of the target molecule in all valid partitions (λ) = −ln (Number of valid partitions − number of positive partitions/Number of valid partitions). The total number of copies of the target molecule in all valid partitions = λ × number of valid partitions. The copies per microliter (copies/µL) can be calculated by λ/V (estimated partition volume) [µL], with results expressed as absolute copies per microliter of reaction volume. The mean copy number of each gene was derived from the QIAcuity software. We express here the mean copy number from 2–3 replicates per cohort.

## 3. Results

### 3.1. Microscopic Examination of Aging Oocysts

**Figure 1:**
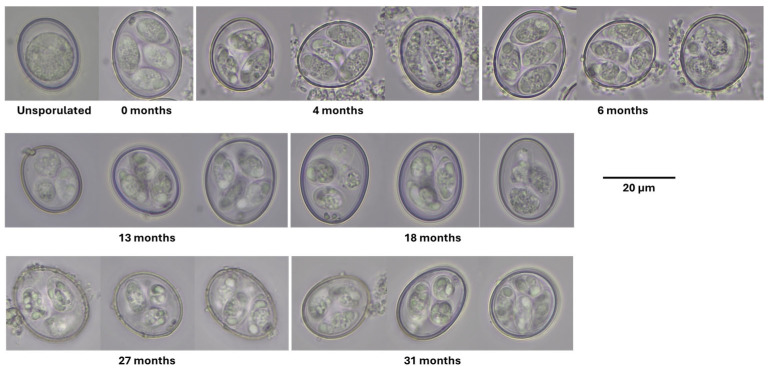
Microscopy of cohorts of aged *E. acervulina* oocysts stored at 4 °C for 0 to 31 months. Oocyst cohorts selected for imaging were approximate to the times that oocysts were held at 4 °C for RNA-Seq. Images of unsporulated and 0-month-old sporulated oocysts display well-defined sporocyst walls and sporozoites. However, as the parasites age, the sporocysts become distorted, and the sporozoites appear less well-defined and accumulate granular structures, particularly in the 4- to 6-month-old oocysts. Oocysts stored for longer periods (from 13 months to 31 months) appear shrunken and degraded; their granularity persisted. A scale bar (20 μm) is provided for reference.

We examined parasite cohorts through time to identify morphological features of senescence. We noticed the accumulation of abnormal oocysts (e.g., containing granular structures, as reported in [12]) as early as 4 months into storage at 4 °C (Figure 1). In almost every sample, oocysts typical in appearance (with non-degraded sporozoites and absence of granular structures) also occurred; however, such typical oocysts decreased in abundance with cohort age. Additionally, sporozoite shrinkage and condensation were evident in aging parasites; after 6 months of storage, sporocyst degradation was also evident (Figure 1). At each time point thereafter, deformities became increasingly evident, e.g., shrunken sporocysts or sporozoites and degraded sporocysts with semi-intact sporozoites. By 18 months, the presence of crystals or amylopectin was pronounced, a hallmark of other aged cohorts.

### 3.2. RNA Quality Assessment Prior to RNA Sequencing

**Figure 2:**
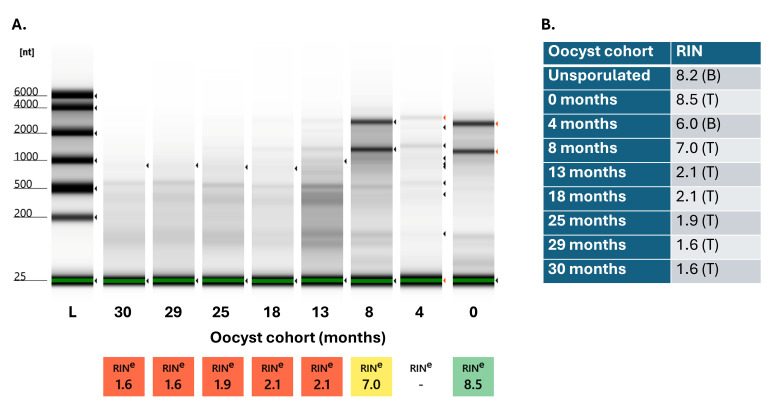
Assessment of the quality of sequenced RNA pools. (**A**). Tapestation results: Left to right— High sensitivity DNA ladder (L) with nucleotide (nt) sizes marked, 30 months, 29 months, 25 months, 18 months, 13 months, 8 months, 4 months, and 0 months. The 4-month sample had an experimental error, so an RNA Integrity Number (RIN) value was not produced by the Tapestation software. An unsporulated oocyst RNA sample was also not part of this image. (**B**). RNA quality (RIN) by Bioanalyzer (B) or Tapestation (T) for cohorts of unsporulated and sporulated oocysts. Note: Unsporulated and 4-month sample Bioanalyzer (B) data are included in the table.

As parasites aged during a period of 4–30 months, RNA integrity decreased, with concomitant decreased cDNA synthesis (an indication of less mRNA). Generally, the quality of RNA diminished markedly in aged cohorts, especially after 8 months (Figure 2). Below, we adopt the conventional language of “expression” and “differential expression” for transcripts whose relative abundance could be measured and changed through time, mindful that some transcripts may have increased their *relative* abundance while undergoing a reduction in their *absolute* expression.

Evidence of minor RNA degradation (RIN ≥ 6) characterized unsporulated oocysts and sporulated oocysts in 0-, 4-, and 8-month cohorts (Figure 2). Much more substantial RNA degradation (RIN < 2) characterized older cohorts. These older cohorts produced poorer-quality double-stranded cDNA sequencing libraries. High concentrations of adapter sequences in the cohorts of 13, 18, 25, 29, and 30 months were present, requiring further purification steps and reducing yield.

### 3.3. RNA-Seq of Aging E. acervulina Cohorts Identified Transcripts Abundant in All Cohorts, as Well as Genes Contributing Especially to Young or Old Cohorts

RNA sequencing was performed on fresh unsporulated oocysts, and on eight sporulated oocyst cohorts held at 4 °C from 0 to 30 months. Here, we sequenced three replicate libraries from each cohort, generating over 47 million paired-end reads (Table 1) for all replicates. The mean mapped reads per cohort ranged from 1,323,099 ± 330,178 (8 months) to 2,019,706 ± 378,543 (13 months). The percentage of mapped reads among replicates ranged from 77.4 to 93.7% (mean = 88.40 ± 5.09%), generally decreasing as parasites aged to 30 months.

**Figure 3:**
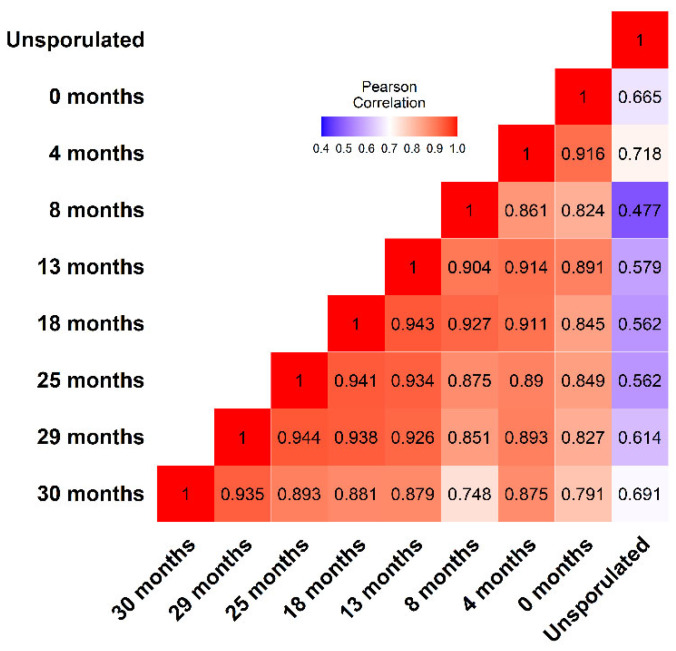
Pearson’s correlation matrix of transcription between unsporulated and aged cohorts of sporulated *E. acervulina* oocysts. Mean log_2_ of mapped reads from three technical replicates per time point was compared for each group.

To estimate the agreement between replicates and groups, we examined the correlation of log_2_-transformed mapped reads. The Pearson’s correlation among replicates within each age cohort (Table S2) was consistently high (*r* > 0.92). Figure 3 shows the correlation of mean log_2_-transformed reads (from three replicates per time point, Table S3). The transcriptome of unsporulated oocysts differed considerably from the transcriptomes of sporulated oocysts, whatever their age. By contrast, the transcriptomes of sporulated oocysts always exhibited correlation ≥ 0.748; cohorts separated by shorter intervals displayed greater correlation in their transcriptomes (Figure 3). Among sporulated cohort pairs, the correlation of mean log_2_-transformed reads ranged from *r* = 0.748 to 0.944; the lowest correlations (*r* < 0.8) in gene expression characterized the comparison of the 30-month cohort with 0- and 8-month cohorts. An erosion of correlation in gene expression through time generally held; typically, a cohort’s gene expression most resembled gene expression at adjacent intervals (e.g., 0 and 4 months or 13 and 18 months). Compared to sporulated oocysts at 0 months, a pronounced drop (*r* from 0.916 to 0.824) occurred at 8 months. This dipped below 80% (0.791) by 30 months. Conversely, gene expression in aging cohorts increasingly resembled the transcriptome of the oldest cohort examined. Thus, the transcriptomes estimated from these libraries exhibited clear and generally consistent temporal evolution.

### 3.4. Expression Analysis

We employed transcripts per million (TPM) to track the relative abundance of ~6900 annotated *E. acervulina* transcripts (Table S4, mean TPM and SD). This mirrors and augments our prior studies of transcription in *E. acervulina* and related species. Here, we first compared each cohort by TPM to ascertain any major underlying differences in transcript abundance, examining those genes exceeding thresholds of 1000 TPM and 100 TPM (as in previous studies) (Table 2). The number of genes exceeding these thresholds was consistent in all cohorts; as in previous studies, most of the annotated genes contributed relatively little to the transcriptome (5865 on average, or ~85%, contributed < 100 TPM). This mirrored prior results for *E. acervulina* during the early hours of sporulation [10]. Previous deep transcriptome sequencing (using the Illumina NextSeq) identified some transcripts accounting for as many as 60,000 TPM (by hour 25); thus, such estimates likely reflect biological reality rather than sampling artifacts. Certain genes (discussed below) consistently contributed disproportionately to the total; among these, *EAH_00004110* and *EAH_00015590* consistently did so.

### 3.5. Constitutively Expressed Genes in Aged Oocyst Cohorts

*E. acervulina* oocysts expressed 53 genes > 1000 TPM throughout a 24 h sporulation period that included measuring unsporulated oocysts [10]. This expression threshold identified the top biomarkers of viability; homologs to many of these genes were also highly expressed in *E. maxima* during sporulation [11]. Consistently high expression for certain genes (Table 2) was also evident in the present study.

**Figure 4:**
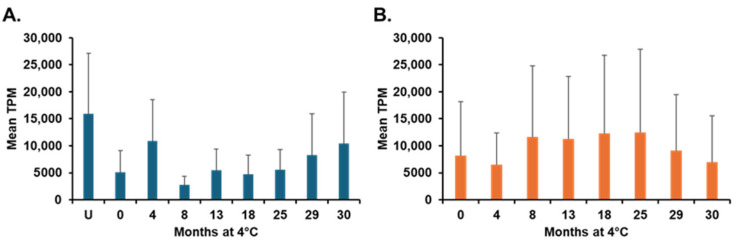
Comparison of mean TPM for constitutively expressed genes identified in aged cohorts of *E. acervulina* oocysts. (**A**). For all cohorts (including unsporulated oocysts), 30 shared genes were identified; their expression was greatest in unsporulated oocysts. (**B**). By excluding unsporulated oocysts, an additional 30 genes contributed > 1000 TPM throughout the 30-month period. TPM was elevated in cohorts stored at 4 °C from 8 to 25 months. The X-axis indicates the stage and age of the oocysts. Unsporulated oocysts are marked by U, whereas 0 to 30 months represent the age of the sporulated oocysts. The Y-axis indicates the mean TPM. Bars on the graph show the standard deviation of TPM in cohorts.

Thirty genes maintained expression of >1000 TPM throughout this study (including the unsporulated group plus all sporulated cohorts; Table S5). These genes ranged in mean TPM from 1766 ± 588 (*EAH_00008670*) to 24,838 ± 14,275 (*EAH_00004110*). The collective contribution of these 30 genes was greatest in unsporulated oocysts and varied thereafter (Figure 4A). Of these 30 genes, all but four (bolded in Table S5) were also constitutively expressed throughout sporulation in our previous *E. acervulina* study [10]. Three of these four exceptions had highest relative abundance in the oldest cohort; these included *EAH_00027050* (histone H2A, which eclipsed 13,000 TPM) and *EAH_00051000* (thioredoxin, >5000 TPM), possibly indicating their involvement in DNA integrity or stress response.

Remarkably, genes such as *EAH_0004110* (this and its homologs have proved abundant in all previous RNA-Seq studies [10,11]) and certain others (also encoding hypothetical proteins) eclipsed 20,000 TPM in the oldest cohort studied (30 months). Their relative abundance was far greater in older cohorts than in freshly sporulated (0 month) oocysts. On the other hand, transcripts such as *EAH_00000650* (actin) and *EAH_00057690* (profilin) were much more abundant in 0 month sporulated oocysts than in other samples, including unsporulated oocysts.

Some transcripts contributed > 10,000 TPM in unsporulated control oocysts; these included *EAH_00004100* and *EAH_00004110* (both encode cation-transporting ATPases), *EAH_00034270* and *EAH_00037200* (which encode zinc finger DHHC domain-containing proteins), and several genes encoding hypothetical proteins. Interestingly, some genes were highly expressed in unsporulated oocysts, decreased in relative abundance immediately upon sporulating (0 months), and thereafter increased in their relative abundance through 30 months (e.g., *EAH_00001460*, *EAH_00004780*, *EAH_00037050* [hypothetical proteins], and *EAH_00004110*). Indeed, relative abundance of these 30 genes remained high from 8 months on, even after senescence reduced their infectiousness (Figure 4A).

We then focused on sporulated oocysts, identifying a total of 60 genes that contributed > 1000 TPM from 0 to 30 months (Figure 4B). The 30 genes meeting this criterion exclusively in sporulated cohorts are denoted in red in Table S6. Among these, many increased their relative abundance as parasites aged, sometimes over six-fold and eclipsing 60,000 TPM. These highly expressed genes, which had overall mean TPM > 25,000, included *EAH_00002690* (serine protease inhibitor), *EAH_00015590*, *EAH_00022290*, and *EAH_00064490*, which encode hypothetical proteins. For these and other sporulated oocyst genes, expression was especially high between 8 and 25 months (Figure 4B).

We then compared the 60 genes constitutively expressed > 1000 TPM in sporulated oocysts to previous *E. acervulina* RNA-Seq data [10]. Overall, 47/60 (78.3%) were also expressed > 1000 mean TPM in sporulated oocysts in the previous report. The 13 genes not meeting this criterion in the original study (Table S6, bolded) almost completely overlapped with the 30 genes constitutively expressed only in the sporulated oocysts. Therefore, we established strong agreement between this and a previous study [10] of sporulating *E. acervulina* conducted at greater sequencing depth.

### 3.6. Functional Characterization of Constitutively Expressed Genes

The group of 30 constitutively expressed genes in common to unsporulated and sporulated oocysts largely overlap (~87%) the constitutively expressed genes we previously identified in sporulating *E. acervulina* oocysts [10], including *EAH_00004100* and *EAH_00004110* (resembling cation-transporting ATPases in *Eimeria* spp.), and *EAH_00004780* and *EAH_00037050* (hypothetical proteins resembling membrane proteins in other Apicomplexa). These 30 genes include many involved in housekeeping functions; we have discussed their potential functions previously [10]. Overall, 188 GO terms were identified for these 30 genes, implicating a range of functions (Table S7). The highest numbers of GO terms were identified for *EAH_00000650* (actin, *n* = 35), followed by *EAH_00029060* (translation initiation factor SUI1, *n* = 22) and *EAH_00027310* (polyubiquitin, *n* = 19); the relative abundance of these transcripts was mid-tier. KEGG pathways were only identified for *EAH_00000650*, *EAH_00019500*, and *EAH_00029060*; denoting purine metabolism, thiamine metabolism, and nicotinate and nicotinamide metabolism.

The 30 genes constitutively expressed only in sporulated oocysts (Table S8) implicated 93 GO terms. Genes *EAH_00010660* (hypothetical protein, resembling phospholipase/carboxylesterase domain-containing proteins in other *Eimeria* spp.), *EAH_00029580* (AGC kinase), and *EAH_00045490* (hypothetical protein, resembling heat shock proteins in other apicomplexan species) each contributed at least 10 GO terms pertaining to hydrolase activity, phosphorylation/signal transduction, and protein folding/cellular response to heat, respectively. The most abundant transcripts (like *EAH_00002690*, *EAH_00015590*, *EAH_00022290*, *EAH_00049920*, and *EAH_00064490*, with >20,000 mean TPM) either had no associated GO terms or only “membrane”. *EAH_00064490* resembles SAG genes in other *Eimeria* spp. This gene and two other SAG-encoding genes (*EAH_00059200*, *EAH_00059950*) displayed ~7000–13,000 overall mean TPM, indicating these proteins are likely important for sporulated oocysts. The latter two genes had GO terms pertaining to actin filament and profilin binding, indicating SAG’s known involvement in motility and invasion. *EAH_00002690* was associated with serine-type endopeptidase inhibitor activity and extracellular space. Four genes—*EAH_00010660*, *EAH_00017770*, *EAH_00045490*, and *EAH_00029580*—were associated with KEGG pathways that included fatty acid elongation, Glycerophospholipid/Glycerolipid metabolism, Purine/Thiamine metabolism, and Human immunodeficiency virus 1 infection, respectively. Except for *EAH_00010660*, these genes were relatively modestly abundant.

### 3.7. Searching for Variable Genes Among Sporulated Oocysts

**Figure 5:**
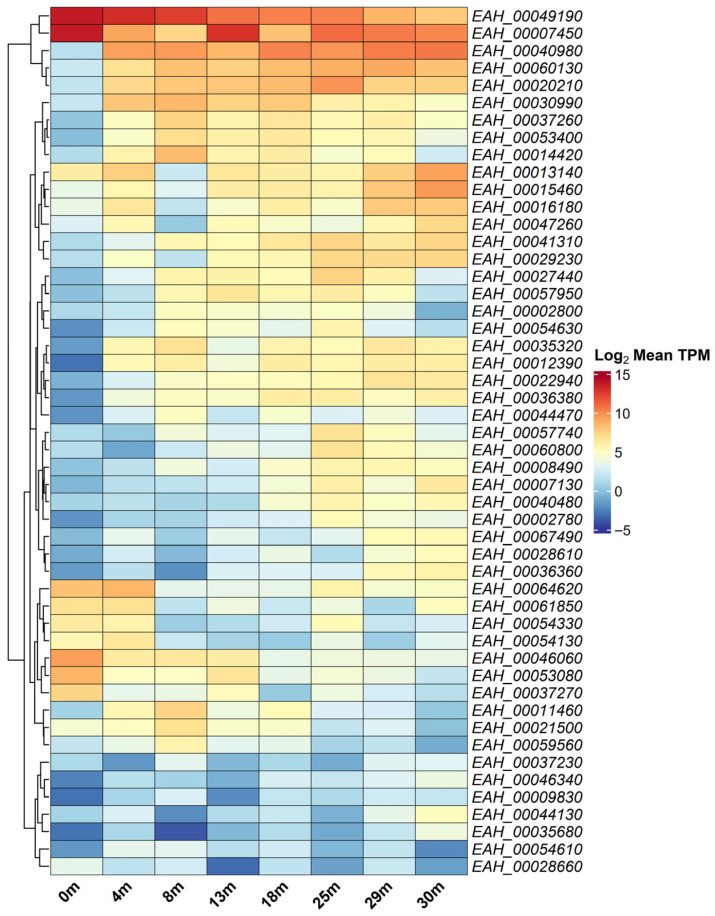
The heatmap displays the 50 genes whose relative abundance changes most through time in aged sporulated oocysts. Most of these genes increase in relative abundance as parasites age; fewer diminish in their relative abundance. Expression variation is displayed for log_2_ mean TPM.

We previously identified patterns of gene expression in sporulating *E. acervulina* oocysts, delineating stage-specific genes by analyzing variance in mean log_2_ TPM. Here, we took similar steps to identify the most variable genes in senescent oocysts. Many of the top 50 most variable genes among cohorts (Figure 5) increased in their relative abundance as oocysts aged (Figure 4); these genes did not, as a rule, contribute especially greatly to the transcript pool.

A small group of genes contributed more to the transcript pool early on, when compared to the 30-month cohort. These included *EAH_00007450, EAH_00046060, EAH_00049190,* and *EAH_00053080*). Their overall mean expression, however, sometimes did not exceed 100 TPM (Table S4).

Taken together, these data make clear that even in older, senescent oocyst cohorts, certain genes contribute disproportionately to the (diminishing) transcript pool. Our experiment could not differentiate ongoing expression from persistence of RNA transcribed when oocysts were young. Senescent and dead sporulated oocysts nonetheless harbor (perhaps express) a repertoire of transcripts that generally predominate in younger parasites.

### 3.8. Differentially Expressed Genes in Live vs. Dead Cohorts

**Figure 6:**
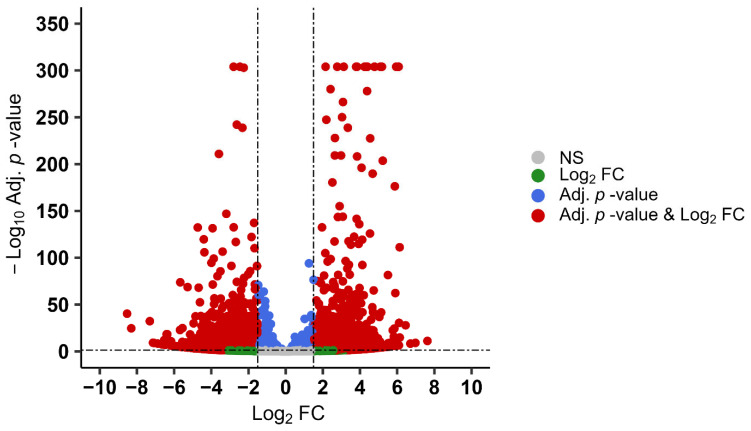
Major differential transcripts that markedly increase, or decrease, in relative abundance after *E. acervulina* oocysts underwent 30 months of storage (compared to those just sporulated). Of 5622 genes whose transcript abundance sufficed to test for statistical significance in expression differences, 2086 (37%) underwent a change of >1.5 or <−1.5 log_2_ Fold Change (FC), with an adjusted *p* < 0.05 (Adj. *p*-value). Of these significant DEGs, 1086 contributed more to the transcriptome at 0 months; 1000 contributed more to the transcriptome after 30 months of storage. The X-axis indicates the log_2_ FC, whereas the Y-axis represents the log_10_ adjusted *p*-value.

We next aimed to identify genes whose changing relative abundance best demarcates the transition from viability to non-viability. We thus sought genes notable for undergoing marked up- or downregulation when comparing their relative abundance at 0 and 30 months. To do so, we established thresholds for differential expression significance and then focused on the most abundant transcripts. Thus, we examined genes that contributed > 1000 TPM at either 0 or 30 months, and which underwent significant differential expression by >1.5 or <−1.5 log_2_ (adjusted *p*-value < 0.05) as estimated using DESeq2. Data sufficed to examine evidence for differential expression among 5622 of the 6867 annotated genes. Of these, 37% (2086) met our criteria for significant differential abundance (Table S9). The volcano plot in Figure 6 depicts these genes, showing 1086 upregulated and 1000 downregulated genes, comparing the transcriptomes of sporulated oocysts after 0 months of storage to those at 30 months.

### 3.9. Major Transcripts Undergoing the Greatest Differential Expression Between 0 and 30 Months

With the established differential expression thresholds, we cross-referenced the highest expressed genes in 0-month oocysts. We identified 86 significant DEGs between 0- and 30-month cohorts that also exceeded 1000 TPM in 0-month sporulated oocysts (Table S10). Interestingly, only two of these 86 (*EAH_00007450*, *EAH_00049190*) were also among the 50 most variably abundant genes discussed above (Figure 5); thus, these genes differing most at the temporal extremes do not typify those undergoing temporal changes throughout the experiment. Indeed, more than 25% of these genes (23 of 86) were constitutively expressed throughout the experiment (as described above).

Of these 86 genes, most (71, 82.6%) exhibited greater relative abundance at month 0 than at month 30, some by more than 6-fold (log_2_) (including *EAH_00059130* (heat shock protein 28) and *EAH_00049190* (hypothetical protein)); nonetheless, some genes increased in their relative abundance at intermediate sampling dates. The genes contributing most to the month 0 transcriptome did not, as a rule, undergo the greatest change in relative expression when compared to oocysts stored for 30 months. For example, *EAH_00011300* (hypothetical protein) and *EAH_00059200* (SAG family member) were expressed >30,000 TPM at month 0, but this represented only a 2–2.7-fold (log_2_) increase over their relative abundance at month 30. *EAH_000032920* (hypothetical protein) contributed ~25,000 TPM at 0 months, over 4-fold (log_2_) greater relative expression than at 30 months; this seems notable because we previously found this transcript characteristic of sporulated, but not unsporulated, oocysts of *E. acervulina* [10].

Conversely, 15 genes contributed less to the transcript pool at 0 months than at 30 months. These did not undergo more than 2.8-fold (log_2_) change; many of these were among those contributing the most to the transcript pool throughout storage (Table S10). The most diminished genes were *EAH_00020540* (hypothetical protein) and *EAH_00027050* (histone H2A), which nonetheless maintained > 11,000 TPM in 30-month-old oocysts.

We identified major functional classes among genes upregulated in 0-month oocysts, including stress response (heat shock and accessory proteins [DNAJ protein], peroxisomal catalase, peroxiredoxin, superoxide dismutase), metabolic pathways (lactate dehydrogenase, glyceraldehyde 3-phosphate dehydrogenase, fructose-bisphosphate aldolase, glucose-6-phosphate isomerase, saccharopine dehydrogenase, transaldolase), and invasion/motility/cell recognition (actin, actin depolymerizing factor, micronemes, myosin, SAGs).

Here, heat shock proteins surfaced as important in senescence. Among all 2086 significant DEGs between 0- and 30-month oocysts (without a TPM constraint, Table S9), 20 were annotated as “heat shock”, “hsp”, “peroxi-”, “oxidoreductase”, “superoxide-”, or “thioredoxin”. Of these, 15 (75%) were upregulated in 0-month oocysts compared to 30-month oocysts, but not all had high relative TPM. For example, *EAH_00067370* (heat shock protein) exhibited over 6-fold log_2_ more expression at month 0 than at month 30, but totaled only ~158 TPM. Still, genes such as *EAH_00044990* (Hsp20/alpha crystallin domain-containing protein) and *EAH_00053040* (superoxide dismutase) contributed over 5000 TPM with >4-fold log_2_ more expression than 30-month oocysts. These genes generally decreased in relative expression as parasites aged (Table S4), but some experienced elevated relative expression at 8–13 months after initially falling at 4 months. The five genes experiencing diminished relative expression at 0 months generally did not exhibit strong expression in any cohort; one (*EAH_00007380*, peroxisome biogenesis factor 7) increased its relative expression by >3-fold (log_2_) at 30 months compared to 0 months.

Our Blast2GO functional analysis of all 86 genes found almost 600 GO terms (Table S11), with the higher number of terms attributed to genes already with an informative annotation (e.g., actin, fructose-bisphosphate aldolase, CAM kinase, SAG, ubiquitin-conjugating enzyme e2). Unfortunately, for many highly expressed and upregulated DEGs of interest that encode hypothetical proteins, such as *EAH_00011300*, *EAH_00032920*, *EAH_00049190*, and *EAH_00049200*, we did not discover any additional annotation information. However, hypothetical protein-encoding genes *EAH_00005140*, *EAH_00010150*, *EAH_00029980*, *EAH_00034850*, *EAH_00049210*, *EAH_00051110*, and *EAH_00066440,* which underwent more than 3-fold (log_2_) change in relative expression in 0-month oocysts, had more informative BLAST hits and GO terms. *EAH_00005140*, notable for >25-fold greater TPM in 0 than in 30-month oocysts, resembles an acyl-CoA synthetase/protein in other *Eimeria* and in *C. cayetanensis*. GO terms associated with this gene include fatty acid metabolic processes, fatty acid import/transport, and other terms pertaining to the endomembrane system. *EAH_00049210* resembles tubulin polymerization-promoting protein in *C. cayetanensis* and p25-alpha family proteins in other apicomplexans; it had associated GO terms pertaining to microtubule formation/polymerization. *EAH_00066440* resembles a heat shock protein in *E. tenella* and has GO terms related to heat response and protein folding.

For the smaller group of genes downregulated in 0-month oocysts (which were mostly annotated as hypothetical proteins), some genes warrant mention. *EAH_00029830*, while on the cusp of our DEG threshold, has 16 associated GO terms related to protein targeting and Golgi vesicle fusion/transport. Genes *EAH_00010660*, *EAH_00020540*, and *EAH_00027050* all underwent over ~2.5-fold (log_2_) increases at 30 months compared to 0 months and had 100 BLAST hits. *EAH_00010660* resembles genes in other Apicomplexa species that encode proteins with abhydrolase and phospholipase/carboxylesterase domains and associated GO terms related to depalmitoylation, lipases/hydrolase activity, and membrane-bound organelles. *EAH_00020540* resembles putative membrane proteins in other Apicomplexa species; its GO terms pertain to chromatin remodeling and transcriptional regulation. *EAH_00027050* encodes a histone H2A protein; its GO terms include chromosome organization and ER/Golgi organelles and transport.

### 3.10. Major Transcripts Undergoing the Greatest Increase in Relative Abundance Through Time

We then identified 66 transcripts contributing > 1000 TPM at month 30 that underwent significant change in their relative abundance at month 30, compared to freshly sporulated oocysts at month 0 (Table S12). These genes generally followed a steady increase in relative abundance from 0 to 30 months (with some exceptions, e.g., spikes at 25, 29 months). Differential expression ranged from 8.53 to −4.24 log_2_ FC. In this analysis, 55/66 (~83%) genes were relatively more abundant at month 30 than at month 0. All genes that were downregulated in 0-month oocysts described above (*n* = 15) were upregulated in 30-month oocysts, as these genes exceeded 1000 TPM in both cohorts. These 15 genes were among those maintaining high expression throughout (discussed above).

Other genes undergoing > 3 log_2_ FC in month 30, compared to month 0, only contributed 1000–3000 TPM at month 30. Many genes undergoing increases in relative abundance at month 30 did not contribute > 1000 overall mean TPM during the time course. Thus, these did not generally typify constitutively expressed genes. Notwithstanding their modest relative abundance throughout the experiment, some experienced an increase in relative TPM (by month 30) of more than 20-fold. For example, *EAH_00040980* (hypothetical protein) underwent ~8.5 log_2_ FC, with >500-fold higher TPM at month 30 than at month 0.

Notably, several genes experiencing the greatest increase in their contribution to the transcript pool at 30 months encode ribosomal proteins. In fact, six genes (*EAH_00003140*, *EAH_00003190*, *EAH_00007090*, *EAH_00007170*, *EAH_00008630*, and *EAH_00021530*) that encode 40S and 60S ribosomal subunits underwent over 3 log_2_ FC, although they seldom topped 1000 TPM in younger cohorts. However, these genes did figure much more prominently at 30 months than at 0 months, based on TPM.

Our functional analysis of transcripts with especially great differential expression in 30-month oocysts (Table S13) identified those that increased in their relative abundance and contributed < 1000 TPM in 0 month-oocysts. This included genes encoding 40S and 60S proteins (with GO terms relating to translation, ribosome binding, regulation of cell processes, and others). The DEG experiencing the greatest increase in relative abundance at 30 months (*EAH_00040980*, ~8.5 log_2_ FC) encodes a hypothetical protein resembling many hypothetical/uncharacterized proteins in other Apicomplexa species but also a chromosome I protein in other *Eimeria* spp. Its GO terms relate to macromolecular metabolism. *EAH_00006500* (~4 log_2_ FC) encodes a hypothetical protein lacking any informative BLASTP match. Its GO terms pertain to mRNA processing, decay, splicing, and transport. *EAH_00024860* (~3 log_2_ FC) encodes an SNF7 family domain protein with associated GO terms of endosome/vesicular transport and protein transport. *EAH_00023750* (DNA repair protein) has over 22 associated GO terms pertaining to DNA damage and binding and meiotic/mitotic processes.

Other interesting genes of note included *EAH_00020350* (haloacid dehalogenase-like hydrolase domain-containing protein), which we previously identified as more abundant in unsporulated than in sporulated oocysts (cite-10). *EAH_00048600* (CHCH domain-containing protein) and *EAH_00053550* (hypothetical protein) eclipsed 5000 TPM in 30-month oocysts, each undergoing 2.7–2.8 fold (log_2_) increases in relative abundance. *EAH_00048600* is associated with GO terms related to mRNA splicing and mitochondria. *EAH_00053550* resembles a membrane-anchored ubiquitin-fold protein in *T. gondii*. *EAH_00003680* (ubiquitin/ribosomal protein CEP52 fusion protein) and *EAH_00010940* (BTB/POZ domain-containing protein) are associated with GO terms pertaining to regulation of proteolysis and ubiquitination. *EAH_00005240* (hypothetical protein) had GO terms related to protein folding and ER. Together with the aforementioned *EAH_00023750* and *EAH_00027050*, these genes appear to maintain basic cellular/repair functions and activate the unfolded protein response in dead or dying parasites.

The relative abundance of genes encoding ribosomal protein subunits in aged oocysts of *E. acervulina* is noteworthy given the abundance of these proteins in other aging and dying organisms. We therefore looked more broadly among the 2086 significant DEGs, searching for related ribosomal protein-encoding genes. We found 53 genes annotated with terms “ribosomal” or “ribosome protein” (Table S9). Interestingly, all but two contribute more to the transcript pool in 30-month oocysts than to the transcript pool in 0-month oocysts. Although few surpassed 1000 TPM, many underwent more than a 3–4 fold (log_2_) increase in relative expression, compared to 0-month oocysts. As with other significantly DEGs, these genes tended to increase their relative abundance as parasites aged, often reaching peak TPM at 29 or 30 months.

Eleven of the 66 DEGs that contributed at least 1000 TPM at month 30 and underwent significant changes in relative abundance contributed less to the transcript pool at month 30 than at month 0. Their relative abundance decreased ~2–4 fold [log_2_]. Among these were some of the most abundant transcripts at month 0, and TPM for some genes surpassed 10,000 in aged cohorts. (Table S12). It is remarkable that such genes so highly expressed in fresh oocysts maintained a relatively high abundance throughout prolonged storage at 4 °C.

### 3.11. qPCR and dPCR to Validate RNA-Seq Using Selected Genes in 0- and 30-Month Oocyst Cohorts

We aimed to investigate the expression of some genes that exhibited different expression profiles in aging oocysts. We selected three genes—*EAH_00004110*, *EAH_00020350*, and *EAH_00049190*—to validate, via qPCR and dPCR, estimates of transcript abundance derived from RNA sequencing. Diminishing RNA quality (or mRNA remaining) in older oocyst cohorts precluded accurate estimation of log_2_ fold changes in gene expression when using total RNA as template for first-strand cDNA synthesis. We observed that RNA quality decreased after 8 months; however, we were still able to create cDNA libraries for sequencing and estimate the abundance of transcripts. Therefore, we utilized double-stranded cDNA sequencing libraries as templates in qPCR and dPCR to validate RNA-Seq. *EAH_00004110* was one of the highest expressed genes in all cohorts, contributing at least 6000 TPM through senescence (constitutively expressed), and it was relatively more abundant (by ~2.3 [log_2_]) at 30 months than at 0 months. Our prior RNA-Seq work with *E. acervulina* showed this gene to be highly expressed during sporulation, and we validated estimates of this gene’s expression by qPCR [10]. *EAH_00020350* was on the lower expression spectrum but increased its expression generally from 0 to 30 months. RNA-Seq estimated it to contribute more to the 30-month transcript pool (by ~3.6 fold (log_2_) than to the 0-month transcript pool. *EAH_00049190* had high expression in all cohorts but decreased expression generally from 0 to 30 months. RNA-Seq estimated it to contribute more to the 0-month transcript pool (by ~6-fold (log_2_)) than to the 30-month transcript pool.

**Figure 7:**
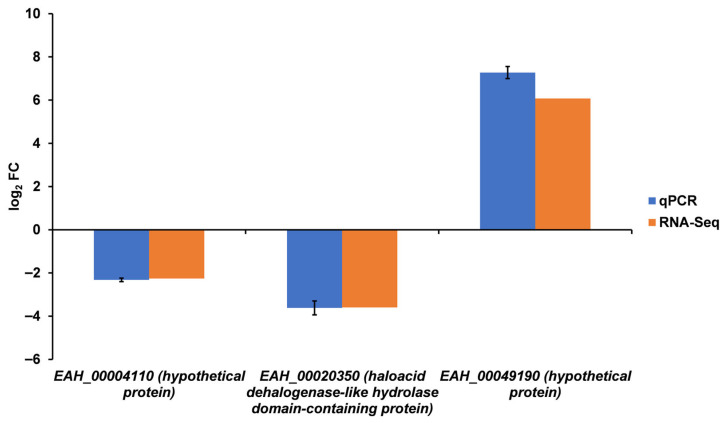
Relative expression of selected genes correlates highly between RNA-Seq and qPCR assays for comparing 0- and 30-month-old oocysts. The log_2_ fold change (FC) using qPCR and RNA-Seq (differential expression by DESeq2) is compared between the two cohorts: genes are up- or downregulated in 0-month vs. 30-month oocysts. Error bars depict standard deviation among mean qPCR estimates from three replicate trials that each employed three technical replicates per cohort.

For relative expression analysis, we assayed cDNA libraries from 0- and 30-month oocyst cohorts in qPCR to determine expression relative to Beta tubulin. For each gene, qPCR estimates of log_2_ FC expression closely approximated that determined from RNA-Seq (Figure 7).

**Figure 8:**
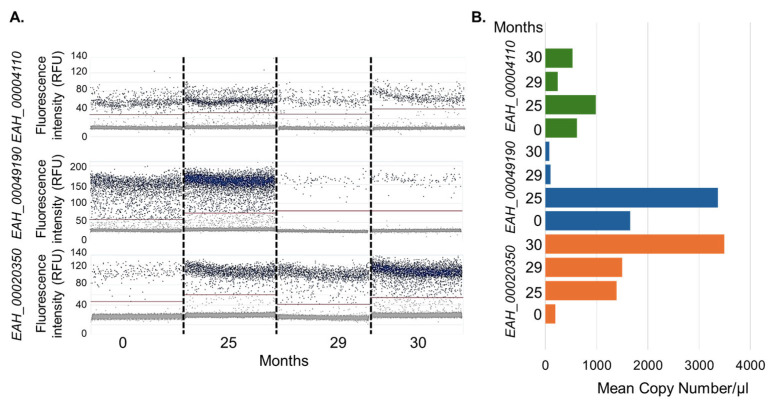
Estimation of absolute gene expression using digital PCR across various aged cohorts. (**A**). Digital qPCR data obtained for genes *EAH_00004110*, *EAH_00020350*, and *EAH_00049190* using double-stranded cDNA sequencing libraries as templates. The absolute expression is represented for three replicates each at 0, 25, 29, and 30 months, displayed from left to right in each gene graph. The X-axis indicates the fluorescence intensity of each nano-partition, with each dot representing a nano-partition. (**B**). Mean copy number calculations for each gene at 0, 25, 29, and 30 months. Each experiment was conducted in three replicates. The fluorescence intensity values of nano-partitions were converted into mean copy numbers/μL using QIAcuity software.

To gain a clearer understanding of the absolute expression of genes throughout the aging process, we employed dPCR. The digital PCR data for these three genes mirrored the findings from RNA-Seq, showing consistent expression trends: *EAH_00049190* demonstrated a decreasing trend, *EAH_00004110* showed high and stable expression, and *EAH_00020350* had an increasing expression trend despite being overall less abundant (Figure 8). Moreover, these results aligned closely with the qPCR data obtained using cDNA sequencing libraries as templates, confirming the expression of certain genes even in 30-month-old oocysts.

## 4. Discussion

Our past RNA-Seq work using *Eimeria* surrogates [10,11] focused on identifying viability biomarkers in developing oocysts. In three species (*E. acervulina*, *E. maxima,* and *E. tenella* [unpublished data]), we demonstrated that particular genes undergo increased relative expression during the progression from unsporulated to sporulated (infectious) oocysts. Here, we extended this approach to identify highly expressed *E. acervulina* genes that undergo changes in their relative abundance through 30 months of storage at 4 °C.

Distinguishing viable from non-viable coccidian oocysts could find broad application. For *Eimeria,* such tools could verify efficacious oocyst cohorts intended for use as vaccines. For *Cyclospora cayetanensis,* no existing diagnostic assay distinguishes viable from dead parasites. Briefly, the U.S. Food and Drug Administration (FDA) established a TaqMan qPCR method targeting the 18S rRNA gene to identify *C. cayetanensis* in fresh produce [29,30,31,32]. This method has been validated in multiple laboratories [33,34], and its use has been extended to other matrices [35,36,37,38,39,40]. However, non-specificity of these assays necessitated development of additional assays, mostly focusing on mitochondrial targets [41,42,43,44]. Neither diagnostic assay differentiates viable, infectious oocysts from the DNA of dead parasites contaminating food or water. An assay that incorporates a biomarker of viability would help assess risk, greatly complementing current diagnostic targets.

We accomplished this study despite notable degradation in the yield and quality of RNA achievable from aging oocyst cohorts (which decreased greatly after 8 months of storage at 4 °C), and despite the limitation this placed on the depth of sequencing. Over time, oocyst cohorts degraded to the point that total RIN values were <2. Therefore, intact mRNA convertible to cDNA during library preparation was very low, and only sub-picomolar library concentrations could be achieved for some cohorts. This limited us to sequencing libraries on an instrument (MiSeq) that tolerates lower library concentrations than required by higher-depth sequencing instruments necessary to assess the contributions of rare transcripts. Recommendations for mRNA sequencing vary. The Encyclopedia of DNA Elements project [45], which focuses on identifying functional elements in human and mouse genomes, suggests 20–30 million aligned paired-end reads suffice to evaluate transcriptional profiles of polyA+ mRNA selected samples. However, Illumina documentation suggests 5–25 million reads/sample suffice to identify highly expressed genes [46].

In this study, sequencing yielded at least 1 million paired-end reads/replicate within a cohort, achieving high correlation among replicates and detecting many genes undergoing significant changes in relative abundance. The more limited scale of a MiSeq arguably compromised estimates of the abundance of scarce transcripts, but consistency across technical replicates substantiated the stability of estimates of the identity and relative abundance of major transcripts. In our prior experience, insights derived from this modest scale strongly predicted results subsequently achieved via deep sequencing (e.g., NextSeq 500 [10] or NextSeq 2000 [11]). Furthermore, these data agree strongly with previous discovery of constitutively expressed and over-expressed genes in sporulated oocysts of *E. acervulina* subjected to deep sequencing [10]. Studies in other systems showed that MiSeq scale sequencing suffices to produce robust estimates of gene expression, even from formalin-fixed paraffin-embedded tissues [47,48,49,50]. Although deeper sequencing coverage enables more precise estimation of the contribution of minor transcripts, the reproducibility of our estimates (across replicates and across studies) substantiated our belief that these estimates reflect biological realities for the major transcripts herein identified.

Our results found that senescent parasites (which we showed to be non-infectious in a previous study [13]) either still maintain active transcription or possess persistent mRNA that could still be transformed to cDNA and sequenced. Oocysts stored at 4 °C for 29 or 30 months appeared to have reduced mRNA, and yet some transcripts that are abundant remain so in storage. These takeaways from our study support this assertion:

- Constitutively expressed genes exhibited high transcript abundance even in cohorts that should be non-infectious in chickens (29, 30 months), on par with cohorts that had been stored at 4 °C for a shorter period. These genes and others are in common with highly expressed constitutively expressed genes in our previous *E. acervulina* RNA-Seq [10]. Many of the highest-expressed genes encode proteins that function in cellular metabolism, perform house-keeping functions, and prepare sporocysts for future invasion (e.g., SAGs). These data reinforce the idea that these genes are likely important markers of sporulation and perhaps viability.
- Fifty-five significantly differentially expressed genes with high abundance were identified in 30-month-old oocysts, and a majority (83%) were upregulated in 30-month vs. 0-month oocysts.
- Genes with increased relative abundance and log_2_ FC in 30-month oocysts encode genes functioning in assorted cellular processes, indicating that dead/dying oocysts activate cellular functions and repair machinery or initiate survival strategies while in storage.
- qPCR validated RNA-Seq for genes that were constitutively expressed in all cohorts (*EAH_00004110*) or more abundant in 30-month-old oocysts (*EAH_0004110, EAH_00020350*).

We cannot state for sure if transcript abundance is due to active transcription of some population of oocysts, but even so, this must be those with the inability to produce infection. All cohorts (save, perhaps, 29 and 30 months) included some metabolically active oocysts (remaining infectious even after years of storage). Nonetheless, an inoculum of 150,000 oocysts of *E. acervulina,* stored at 4 °C for 27 months, still sufficed to establish infection [13]; we do not know how many such survivors are required to achieve that outcome, but no infection was observed after 30 months of refrigerated storage. Older cohorts accumulated visibly dead and dying oocysts, as our morphological observations showed an accumulation of deformed and granule-containing structures within oocysts through time. This is consistent with a recent study showing such structures in aged oocysts held at 4 °C, which autofluoresce [12]. Any undegraded RNA in these oocysts would contribute to the libraries we sequenced; our methods did not afford the means to estimate any such contribution. Furthermore, we did not attempt to estimate populations of viable vs. non-viable oocysts in each cohort prior to RNA-Seq. Therefore, we acknowledge the possibility that some population of viable oocysts exists in all cohorts. Studies in *Cryptosporidium parvum* showed gradual degradation of some gene encoding mRNAs (e.g., β-tubulin) in heat-shocked oocysts and oocysts stored for prolonged periods at 4 °C [51,52], but transcripts persist and can still be detectable in infectious oocysts stored for 39 weeks. We did not include internal control groups (e.g., a transcription inhibition control) that could have helped explain if high transcript abundance is due to resistance to degradation or persistent active transcription during oocyst aging. Such an experiment would be interesting to include in the future to facilitate clarifying this matter.

A major goal of this work was to identify viability markers by interrogating differences in gene expression between the newest and oldest sporulated oocysts. A major takeaway for upregulated DEGs at 0 months was that the more abundant transcripts did not necessarily undergo the greatest change in relative expression when compared to oocysts stored for 30 months. Of particular interest was *EAH_000032920* (hypothetical protein), which contributed ~25,000 TPM at 0 months, over 4-fold (log_2_) greater relative expression than at 30 months. We previously reported this gene was highly expressed in sporulated *E. acervulina* oocysts [10], and its likely homolog in *E. maxima* (*EMWEY_00001470* = hypothetical protein) was highly differentially expressed in sporulated oocysts of two strains of *E. maxima* compared to unsporulated oocysts [11]. The relative abundance of *EAH_00032920* in sporulated oocysts exceeded that in unsporulated oocysts by 10-fold log_2_ in these previous studies; here, too, its expression in newly sporulated oocysts exceeded its expression in unsporulated oocysts to a similar degree. *EAH_00032920* displayed a progressive decrease in expression in sporulated oocysts through 30 months of storage at 4 °C, but its relative predominance in the transcript pool never dipped below ~1400 TPM. Therefore, these transcripts are likely important to sporulated *Eimeria* spp. oocysts and hold promise as markers to age sporulated oocysts; we wish to investigate these further in related species, including *C. cayetanensis*.

Our functional analysis of upregulated DEGs in 0-month oocysts revealed several categories, including those with stress response functions (heat shock and accessory proteins, peroxisomal catalase, peroxiredoxin, superoxide dismutase). Although not always contributing the greatest TPM, these genes were sometimes differentially expressed more than 4-fold (log_2_). Genes encoding stress response functions generally decreased in relative expression as parasites aged and were <1000 TPM by 30 months. Furthermore, genes encoding enzymes in metabolic pathways and invasion/motility/cell recognition were also upregulated at 0 months. Yet, aged oocysts still maintained high abundance of genes like *EAH_00057690* (profilin family protein) and *EAH_00059200* (SAG family member) that, although expressed >20,000 TPM at month 0, still had at least 3400 TPM at month 30. Some of these genes previously proved highly differentially expressed and upregulated in our previous studies of *E. acervulina* and *E. maxima* sporulating oocysts [10,11]; thus, their presence is likely normal in demarcating mature oocysts. The decreased abundance of heat shock proteins (HSPs) in senescent oocysts may be a signal in a population of mostly non-viable oocysts. HSPs are expressed more highly in sporulated oocysts of multiple apicomplexan species, including *Eimeria*, *Plasmodium*, and *Toxoplasma* [10]. Since they may play roles in parasite development, survival, and stress response, it stands to reason that aging oocysts lack expression of these genes that factor in maintaining viability. Interestingly, we found that some downregulated transcripts at 0 months exceeded 1000 TPM at both 0 and 30 months. Many genes encode hypothetical proteins that function in cellular transport/protein processing, whereas others pertain to histones and chromatin changes. Together, these data show that groups of stress response and other metabolic and cellular proteins are normally expressed in viable, sporulated oocysts and decrease their relative abundance markedly in non-viable oocysts. By contrast, transcripts involved in critical cellular activities such as endomembrane systems and chromatin remodeling remain at high relative abundance in the oldest oocysts. Organelle transport and chromatin remodeling may be upregulated processes important to eukaryotic senescence and reflected in the transcript pool for survival or alteration of gene expression profiles [53,54,55] (although not necessarily representing infectious oocysts).

Focusing on upregulated DEGs in 30-month oocysts, often the greatest transcript abundance was for genes with moderate to low differential expression. A major finding was that several of the upregulated 30-month oocyst genes encode ribosomal proteins. Six genes encoding 40S and 60S ribosomal subunits had > 3 log_2_ FC compared to 0-month oocysts, with very low TPM in 0-month oocysts. A more global analysis within the total of significant DEGs found 53 genes with likely ribosomal function, and almost all had higher TPM in 30-month oocysts. The increased abundance of ribosomal protein-encoding transcripts may be due to their inherent stability, even at cold temperatures [56], and need to assemble the large and rigid ribonucleoprotein complex for translation. *C. parvum* oocysts stored at room temperature or exposed to heat maintain 18S rRNA transcription for longer periods compared to other transcripts [51]. Alternatively, the persistence of ribosomal unit-encoding mRNAs may represent a shift towards basic cellular processes or integrated stress response [53], or the differential persistence of ribosomal subunits as the pool of messenger RNA degrades as metabolism and transcription diminish. Recent work reported *P. falciparum* degrades ribosomal RNA and switches rRNA types during its development, which could be important for regulating quiescence [57].

## 5. Conclusions

This study of *E. acervulina* oocysts stored at 4 °C provides a starting point for understanding what coccidian parasites may undergo during refrigerated conditions typical of produce packing, shipping, and storage. Warmer and more variable environmental conditions likely hasten senescence and non-viability [13]. Such knowledge would be valuable for monitoring *Eimeria* oocyst viability in the poultry industry and could be translatable to *Cyclospora* environmental diagnostics and control. Given the global effects of coccidiosis on poultry production and cyclosporiasis outbreaks in humans, it is more important than ever that we have reliable detection tools. Although we have recently developed microscopic and artificial intelligence–based tools to discriminate viable from non- -viable oocysts, there are currently no molecular biomarkers capable of distinguishing live from dead oocysts, which presents a serious challenge for ensuring *Eimeria* vaccines elicit adequate protective immunity in chickens. By the same token, cyclosporiasis diagnostics cannot accurately assess the risk of infection from oocysts detected on produce or in water.

Our data provide genes to validate as potential markers for viable oocysts and prompt further studies, including evaluating panels of *Eimeria* genes by more sensitive and rapid assays such as dPCR. dPCR may detect and quantify known and low abundant transcripts, including those that have high log fold changes in live vs. dead oocysts. Furthermore, the experiments here provide valuable surrogate data for *C. cayetanensis,* which is harder to obtain, more dangerous to manipulate, and slower to mature. Ultimately, the methods elaborated using *Eimeria* surrogates should prove useful in illuminating sporulation and senescence in *Cyclospora* via direct empirical tests. Homologous transcripts in *C. cayetanensis* are accessible from published genomic data, and similar methods could be applied for viability studies of oocysts in contaminated food and water sources. Produce growers, public health agencies, and other regulators would clearly benefit from novel tools capable of sensitive and efficient detection of viable *C. cayetanensis*.

## Figures and Tables

**Table 1:** Summary and descriptive statistics of RNA-Seq data for different-aged groups of *E. acervulina* oocysts.

Time (Months Held at 4 °C)	Replicate *	Reads	Mapped Reads	Mapped Reads (%)	Mean Mapped Reads per Triplicate	Mapped Reads SD per Triplicate
Unsporulated	r1	1,697,936	1,541,043	90.8%		
	r2	2,369,306	2,158,594	91.1%		
	r3	1,621,188	1,475,095	91.0%	1,724,911	377,025
0	r1	1,056,156	989,227	93.7%		
	r2	1,428,778	1,338,083	93.7%		
	r3	1,922,402	1,800,033	93.6%	1,375,781	406,715
4	r1	1,932,934	1,769,283	91.5%		
	r2	1,577,872	1,447,357	91.7%		
	r3	1,154,220	1,059,317	91.8%	1,425,319	355,496
8	r1	1,768,566	1,646,156	93.1%		
	r2	1,063,808	986,234	92.7%		
	r3	1,441,558	1,336,908	92.7%	1,323,099	330,178
13	r1	2,693,428	2,455,852	91.2%		
	r2	2,000,036	1,776,589	88.8%		
	r3	2,016,682	1,826,678	90.6%	2,019,706	378,543
18	r1	1,686,672	1,496,423	88.7%		
	r2	1,423,034	1,298,344	91.2%		
	r3	2,069,778	1,602,892	77.4%	1,465,886	154,553
25	r1	1,331,836	1,162,816	87.3%		
	r2	1,622,294	1,407,671	86.8%		
	r3	1,858,120	1,583,341	85.2%	1,384,609	211,209
29	r1	1,848,712	1,532,820	82.9%		
	r2	2,321,444	1,935,628	83.4%		
	r3	1,741,658	1,547,683	88.9%	1,672,044	228,392
30	r1	1,803,484	1,451,531	80.5%		
	r2	2,296,274	1,817,005	79.1%		
	r3	1,400,300	1,083,828	77.4%	1,450,788	366,589

* RNA-Seq was performed on three technical replicates (designated r1, r2, r3) from each group.

**Table 2:** RNA-Seq expression analytics for *E. acervulina* aged oocyst cohorts.

Time (Months)	Abundance of Most Transcribed Gene (TPM *)	Most Transcribed Gene	Number of Genes				
			TPM >1000	TPM >100	TPM <100	Upregulated **	Downregulated **
Unsporulated	42,895	*EAH_00004110*	105	926	5941	1757	1011
0	42,112	*EAH_00059200*	143	1011	5856	N/A	N/A
4	32,552	*EAH_00004110*	126	1036	5831	602	537
8	51,548	*EAH_00022290*	160	1089	5778	1146	934
13	42,713	*EAH_00015590*	140	957	5910	647	584
18	54,352	*EAH_00022290*	139	1024	5843	828	745
25	60,279	*EAH_00015590*	129	976	5891	618	879
29	44,470	*EAH_00015590*	134	998	5869	890	913
30	45,204	*EAH_00004110*	128	1001	5866	1086	1000

* Transcripts per million (TPM), averaged among technical replicates, for each group. Unsporulated oocysts were collected prior to sporulating oocysts for 24 h (0 months). ** Genes expressed significantly more, or less in aged sporulated cohorts, than at 0 months; pairwise differential expression thresholds of >1.5 or <-1.5 log_2_ fold change (FC) and adjusted *p*-value < 0.05.

## Data Availability

The data discussed in this publication have been deposited into NCBI’s Gene Expression Omnibus (GEO) [58] and are accessible through GEO Series accession number GSE306186 and https://www.ncbi.nlm.nih.gov/geo/query/acc.cgi?acc=GSE306186.

## Supplementary Materials

The following supporting information can be downloaded at: https://www.mdpi.com/article/10.3390/microorganisms14051116/s1, Table S1. Oligonucleotides used for qPCR and dPCR in this study. Gene names and annotated protein descriptions (columns A,B), 5’-3’ oligonucleotide sequence (column C), and suggested final concentration (nM, nanomolar) for use (column D) are included. Sequences for most genes were previously reported in [10]; sequences for genes *EAH_00020350* and *EAH_00049190* are novel to this work. Table S2. Log_2_ mapped reads for replicate samples of unsporulated and sporulated *E. acervulina* oocyst cohorts in this study. Included are the *E. acervulina* gene name (column A) and log_2_-tranformed reads per cohort (three replicates per time point)—Unsporulated (columns E–G), 0 months (columns H–J), 4 months (columns K–M), 8 months (columns N–P), 13 months (columns Q–S), 18 months (columns T–V), 25 months (columns W–Y), 29 months (columns Z–AB), and 30 months (columns AC–AE). Additionally, Pearson product correlations of mean log_2_ mapped reads between cohort replicates are displayed in columns AH–BI. Table S3. Mean log_2_ mapped reads for unsporulated and sporulated *E. acervulina* oocyst cohorts in this study. Included are the *E. acervulina* gene name (column A), annotated protein description (column B), nucleotide sequence (column C), amino acid sequence (column D), mean log_2_ (from three replicates) mapped reads for each study cohort—Unsporulated, 0 months, 4 months, 8 months, 13 months, 18 months, 25 months, 29 months, 30 months (columns E–M). Table S4. Mean and standard deviation (SD) for transcripts per million (TPM) for unsporulated and sporulated *E. acervulina* oocyst cohorts in this study. Included are the *E. acervulina* gene name (column A), annotated protein description (column B), nucleotide sequence (column C), amino acid sequence (column D), mean TPM per cohort (from three replicates)—Unsporulated (columns E), 0 months (column F), 4 months (column G), 8 months (column H), 13 months (column I), 18 months (column J), 25 months (column K), 29 months (column L), and 30 months (column M). The corresponding SD of TPM per cohort is given in columns O–W. Table S5. Constitutively expressed genes shared by unsporulated and sporulated *E. acervulina* oocyst cohorts in this study. Included are the *E. acervulina* gene name (column A), annotated protein description (column B), nucleotide sequence (column C), amino acid sequence (column D), mean transcripts per million (TPM) per cohort—Unsporulated (columns E), 0 months (column F), 4 months (column G), 8 months (column H), 13 months (column I), 18 months (column J), 25 months (column K), 29 months (column L), and 30 months (column M). The overall mean TPM for each gene (calculated for all cohorts) is displayed in column N, and the corresponding overall standard deviation (SD) of TPM is given in column O. Bolded genes (*n* = 4) did not meet constitutive gene criteria in our previously published *E. acervulina* RNA-sequencing study [10]. Table S6. Shared constitutively expressed genes identified for *E. acervulina* sporulated oocyst cohorts. Included are the *E. acervulina* gene name (column A), annotated protein description (column B), nucleotide sequence (column C), amino acid sequence (column D), mean transcripts per million (TPM) per sporulated oocyst cohort—0 months (column E), 4 months (column F), 8 months (column G), 13 months (column H), 18 months (column I), 25 months (column J), 29 months (column K), and 30 months (column L). The overall mean TPM for each gene (calculated for 0–30 month sporulated cohorts) is displayed in column M, and the corresponding overall standard deviation (SD) of TPM is given in column N. Genes colored in red (*n* = 30) are those unique to sporulated oocysts, as they did not have >1000 mean TPM in unsporulated oocysts. Bolded genes (*n* = 13) did not meet constitutive gene criteria in our previously published *E. acervulina* RNA-sequencing study [10]. Table S7. Functional analysis of constitutively expressed genes shared by unsporulated and sporulated *E. acervulina* oocyst cohorts. Included are the *E. acervulina* gene names and original gene annotations in columns A and B, respectively. The overall mean transcripts per million (TPM) (for all replicates and cohorts in the study) for each gene is listed in column C. BLASTP data include the length of the protein (column D), overall number of BLASTP hits found (column E), hit e-value (column F), the mean percent similarity for hits (column G), BLAST hit description (column H), and best other BLASP hit (column I; not applicable [N/A] if sufficient annotation already existed). Gene Ontology (GO) data include number of GO terms (column J), GO ID numbers (column K; C = Cellular Component, F = Molecular Function, P = Biological Process), and GO term names (column L), Enzyme Commission (EC) number(s) linked to the GO terms of a given sequence (column M), EC enzyme names (column N). InterPro data include ID names (column O), GO ID numbers (column P), and GO names (column Q). KEGG pathways are displayed in column R. Bolded genes (*n* = 4) did not meet constitutive gene criteria in our previously published *E. acervulina* RNA-sequencing study [10]. Table S8. Functional analysis of shared constitutively expressed genes in *E. acervulina* sporulated oocysts cohorts. Included are the *E. acervulina* gene names and original gene annotations in columns A and B, respectively. The overall mean transcripts per million (TPM) (for all replicates and cohorts in the study) for each gene is listed in column C. BLASTP data include the length of the protein (column D), overall number of BLASTP hits found (column E), hit e-value (column F), the mean percent similarity for hits (column G), BLAST hit description (column H), and best other BLASP hit (column I; not applicable [N/A] if sufficient annotation already existed). Gene Ontology (GO) data include number of GO terms (column J), GO ID numbers (column K; C = Cellular Component, F = Molecular Function, P = Biological Process), and GO term names (column L), Enzyme Commission (EC) number(s) linked to the GO terms of a given sequence (column M), EC enzyme names (column N). InterPro data include ID names (column O), GO ID numbers (column P), and GO names (column Q). KEGG pathways are displayed in column R. Bolded genes (*n* = 12) did not exceed 1000 mean TPM in sporulated oocysts in our previously published *E. acervulina* RNA-sequencing study [10]. Table S9. Significantly differentially expressed genes (DEGs) expressed between 0- and 30-month *E. acervulina* sporulated oocysts. Included are the *E. acervulina* gene name (column A), annotated protein description (column B), nucleotide sequence (column C), amino acid sequence (column D), differential expression log_2_ ratio (column E), and adjusted *p*-value (column F). DEGs reported here are genes expressed significantly more when comparing 0- and 30-month oocyst cohorts in DESeq pairwise comparison, meeting thresholds of >1.5 or < −1.5 log_2_ fold change (FC) and adjusted *p* < 0.05. The values here show genes upregulated or downregulated in 0-month oocysts. Table S10. Significantly differentially expressed genes (DEGs) between 0–30 month *E. acervulina* sporulated oocysts, genes >1000 TPM in 0-month oocysts. Included are the *E. acervulina* gene name (column A), annotated protein description (column B), nucleotide sequence (column C), amino acid sequence (column D), mean transcripts per million (TPM) per sporulated oocyst cohort—0 months (column E) and 30 months (column F). The overall mean TPM for each gene (calculated for 0–30-month sporulated cohorts) is displayed in column G. The differential expression (DE) log_2_ fold change and differential expression adjusted (Adj.) *p*-values are shown in columns H and I, respectively. DEGs reported here are genes expressed significantly more in 0–30 months DESeq pairwise comparison, meeting thresholds of >1.5 or <−1.5 log_2_ fold change (FC) and adj. *p* < 0.05 with >1000 mean TPM at 0 months. Table S11. Functional analysis of significantly differentially expressed genes (DEGs) between 0–30 month *E. acervulina* sporulated oocysts, genes >1000 TPM in 0-month oocysts. Included are the *E. acervulina* gene name (column A), annotated protein description (column B), mean transcripts per million (TPM) per sporulated oocyst cohort—0 months (column C) and 30 months (column D). The differential expression (DE) log_2_ fold change and differential expression adjusted (Adj.) *p*-values are shown in columns E and F, respectively. BLASTP data include the length of the protein (column G), overall number of BLASTP hits found (column H), hit e-value (column I), the mean percent similarity for hits (column J), BLAST hit description (column K), and best other BLASP hit (column L; not applicable [N/A] if sufficient annotation already existed). Gene Ontology (GO) data include number of GO terms (column M), GO ID numbers (column N; C = Cellular Component, F = Molecular Function, P = Biological Process), and GO term names (column O), Enzyme Commission (EC) number(s) linked to the GO terms of a given sequence (column P), EC enzyme names (column Q). InterPro data include ID names (column R), GO ID numbers (column S), and GO names (column T). KEGG pathways are displayed in column U. DEGs reported here are genes expressed significantly more in 0–30 months DESeq pairwise comparison, meeting thresholds of >1.5 or <−1.5 log_2_ fold change (FC) and adj. *p* < 0.05 with >1000 mean TPM at 0 months. Table S12. Significantly differentially expressed genes (DEGs) expressed between 0–30 month *E. acervulina* sporulated oocysts, genes >1000 TPM in 30-month oocysts. Included are the *E. acervulina* gene name (column A), annotated protein description (column B), nucleotide sequence (column C), amino acid sequence (column D), mean transcripts per million (TPM) per sporulated oocyst cohort—0 months (column E) and 30 months (column F). The overall mean TPM for each gene (calculated for 0–30-month sporulated cohorts) is displayed in column G. The differential expression (DE) log_2_ fold change and differential expression adjusted (Adj.) *p*-values are shown in columns H and I, respectively. DEGs reported here are genes expressed significantly more in 0–30 months DESeq pairwise comparison, meeting thresholds of >1.5 or <−1.5 log_2_ fold change (FC) and adj. *p* < 0.05 with >1000 mean TPM at 30 months. Table S13. Functional analysis of significant DEGs expressed between 0–30 month *E. acervulina* sporulated oocysts, genes >1000 TPM in 30-month oocysts. Included are the *E. acervulina* gene name (column A), annotated protein description (column B), mean transcripts per million (TPM) per sporulated oocyst cohort—30 months (column C) and 0 months (column D). The differential expression (DE) log_2_ fold change and differential expression adjusted (Adj.) *p*-values are shown in columns E and F, respectively. BLASTP data include the length of the protein (column G), overall number of BLASTP hits found (column H), hit e-value (column I), the mean percent similarity for hits (column J), BLAST hit description (column K), and best other BLASP hit (column L; not applicable [N/A] if sufficient annotation already existed). Gene Ontology (GO) data include number of GO terms (column M), GO ID numbers (column N; C = Cellular Component, F = Molecular Function, P = Biological Process), and GO term names (column O), Enzyme Commission (EC) number(s) linked to the GO terms of a given sequence (column P), EC enzyme names (column Q). InterPro data include ID names (column R), GO ID numbers (column S), and GO names (column T). KEGG pathways are displayed in column U. DEGs reported here are genes expressed significantly more in 0–30 months DESeq pairwise comparison, meeting thresholds of >1.5 or <−1.5 log_2_ fold change (FC) and adj. *p* < 0.05 with >1000 mean TPM at 30 months.

## Abbreviations

The following abbreviations are used in this manuscript:

Adj. *p*-value	Adjusted *p*-value
AI	Artificial Intelligence
ATPase	Adenosine triphosphatase
BLAST	Basic Local Alignment Search Tool
CAMK	Ca2+/calmodulin-dependent protein kinase
cDNA	Complementary DNA
CDS	Coding DNA Sequence
CRIS	Current Research Information System
DEG(s)	Differentially Expressed Gene(s)
DNA	Deoxyribonucleic acid
Dnase	Deoxyribonuclease
EC	Enzyme Commission
eggNOG	Evolutionary genealogy of genes: Non-supervised Orthologous Groups
ENCODE	Encyclopedia of DNA Elements
ER	Endoplasmic Reticulum
FC	Fold change
FDA	Food and Drug Administration
GEO	Gene Expression Omnibus
GO	Gene Ontology
KEGG	Kyoto Encyclopedia of Genes and Genomes
mRNA	Messenger RNA
NCBI	National Center for Biotechnology Information
PCR	Polymerase Chain Reaction
qPCR	Quantitative PCR
RIN	RNA Integrity Number
RNA	Ribonucleic acid
rRNA	Ribosomal RNA
Rnase	Ribonuclease
RNA-Seq	RNA Sequencing
SAG(s)	Surface Antigen(s)
SD	Standard Deviation
TPM	Transcripts Per Million
U.S.	United States
USA	United States of America
USDA	United States Department of Agriculture
